# Consensus Statements among European Sleep Surgery Experts on Snoring and Obstructive Sleep Apnea: Part 3 Palatal Surgery, Outcomes and Follow-Up, Complications, and Post-Operative Management

**DOI:** 10.3390/jcm13185438

**Published:** 2024-09-13

**Authors:** Ewa Olszewska, Andrea De Vito, Clemens Heiser, Olivier Vanderveken, Carlos O’Connor-Reina, Peter Baptista, Bhik Kotecha, Claudio Vicini

**Affiliations:** 1 Department of Otolaryngology, Sleep Apnea Surgery Center, Medical University of Bialystok, 15-276 Bialystok, Poland; 2 Department of Surgery, Morgagni-Pierantoni Hospital, Health Local Agency of Romagna, 47121 Forlì, Italy; 3 Faculty of Medicine and Health Sciences, University of Antwerp, 2000 Antwerp, Belgium; 4 Department of Otorhinolaryngology/Head and Neck Surgery, Klinikum Rechts der Isar, Technical University of Munich, 80333 München, Germany; 5 Department of Otorhinolaryngology, Head and Neck Surgery, Antwerp University Hospital, 2650 Antwerp, Belgium; 6 Hospitales Quironsalud Marbella, 29603 Malaga, Spain; 7 Departmento de Orl, Clinica Universidad da Navarra, 31008 Pamplona, Spain; peterbaptista@gmail.com; 8 Nuffield Health Brentwood, Essex, Brentwood CM15 8EH, UK; bhikkot@aol.com; 9 UME Health, 17 Harley Street, London W1G 9QH, UK; 10 GVM Care & Research ENT Consultant, GVM Primus Medica Center, GVM San Pier Damiano Hospital, 48018 Faenza, Italy; claudio@claudiovicini.com

**Keywords:** snoring, sleep apnea, obstructive sleep apnea, consensus, decision-making, treatment, surgical management, palatoplasty, palatal surgery, outcomes, follow-up, complications, post-operative management, pain management

## Abstract

**Background/Objectives:** Exploring and establishing a consensus on palatal surgery, the outcomes and follow-up after the palatal surgery, the complications of palatal surgery, and the post-operative management after palatal surgery for snoring and obstructive sleep apnea (OSA) among sleep surgeons is critical in the surgical management of patients with such conditions. **Methods:** Using the Delphi method, a set of statements was developed based on the literature and circulated among a panel of eight European experts. Responses included agreeing and disagreeing with each statement, and the comments were used to assess the level of consensus and to develop a revised version. The new version with the level of consensus and anonymized comments was sent to each panel member as the second round. This was repeated over a total of five rounds. **Results:** The final set included a total of 111 statements, 27 of which were stand-alone questions and 21 of which contained 84 sub-statements. Of the 34 statements regarding palatal surgery, consensus was achieved among all eight, seven, and six panelists for 50%, 35.3%, and 5.9% of the questions, respectively. Of the 43 statements regarding the outcomes and follow-up after the palatal surgery, consensus was achieved among all eight, seven, and six panelists for 53.5%, 23.3%, and 4.7% of the questions, respectively. Of the 24 statements regarding complications after the palatal surgery, consensus was achieved among all eight, seven, and six panelists for 91.7%, 0%, and 4.2% of the questions, respectively. Of the 10 statements regarding post-operative management after palatal surgery, consensus was achieved among all eight, seven, and six panelists for 10%, 30%, and 30% of the papers, respectively. **Conclusions:** This consensus provides an overview of the work of European sleep surgeons to develop a set of statements on palatal surgery for the treatment of snoring and OSA, the outcomes and follow-up, the complications, and the post-operative management of palatal surgery. We believe that this will be helpful in everyday practice. It also indicates key areas for further studies in sleep surgery.

## 1. Introduction

The surgical management of snoring and obstructive sleep apnea (OSA) is inherently complex and multifaceted, requiring careful consideration of different factors. These include the patient’s clinical history, age, and comorbid conditions, as well as detailed anatomical assessments of the upper airways and craniofacial structure. The majority of patients with OSA complain of excessive daytime sleepiness and impulsivity. A detailed anatomical assessment of the upper airways and craniofacial structure is crucial in the diagnostic process of OSA patients [1]. A thorough evaluation encompasses identifying the specific sites of obstruction, the severity of snoring, and the staging and severity of OSA.

Moreover, recent advancements emphasize the importance of phenotyping and endotyping OSA and characterizing the phenotypes of the palate. Diagnostic procedures, such as drug-induced sleep endoscopy (DISE) and advanced imaging techniques of the upper airways, play a crucial role in pinpointing the precise anatomical and functional abnormalities contributing to the condition.

Individual patient factors, including their response to and compliance with non-surgical treatments, significantly influence the decision-making process. Additionally, patient expectations and preferences must be taken into account to ensure that the chosen surgical intervention aligns with their lifestyle and treatment goals. The array of available surgical options further necessitates a personalized approach to optimize outcomes for each patient.

These comprehensive evaluations and tailored approaches underscore the complexity and individualized nature of surgical decision-making in the management of snoring and OSA.

The soft palate has been the focus of attention regarding snoring and OSA for decades [2] and has been blamed as the site of vibrating tissues during sleep, causing snoring [3,4]. Similarly, the soft palate is considered one of the main sites of upper airway obstruction leading to upper airway collapse during sleep, manifesting as OSA. Subsequently, the soft palate has been the primary target for the management of snoring and OSA [5].

Surgical interventions on the soft palate have been the most common surgical procedure for managing snoring and OSA [6]. The first surgical methods to treat snoring appeared in the literature in 1955 [7]. Then, uvulectomy was introduced as a method to treat snoring in the 1960s. This reduced the intensity of snoring but not its frequency [4]. Since Ikematsu introduced the first uvulopalatopharyngoplasty (UPPP) for snoring in 1964 [2], several palate-based techniques have been developed to treat snoring and OSA. UPPP procedures started in 1981 when Fujita introduced UPPP and demonstrated the postoperative results of 12 patients, showing an improvement in eight of them on a polysomnogram [8].

Surgical techniques for the management of snoring and obstructive sleep apnea (OSA) have significantly evolved. This was driven by expanding knowledge on the functional anatomy of the upper airways, the mechanisms underlying upper airway obstruction, and the pharyngeal patterns of collapse. This deeper understanding has facilitated the development of more targeted and effective surgical interventions, tailored to address specific anatomical and functional abnormalities contributing to OSA [8,9].

Surgical interventions in OSA patients have evolved from ablative methods, such as radical UPPP, to more functional and less invasive ones, such as expansion sphincterpharyngoplasty (ESP), barbed stitch reposition pharyngoplasty (BRP), and the Australian modification of uvulopalatopharyngoplasty [9,10,11,12]. The selection of the palatoplasty method for an individual patient has not been standardized. Surgeons may select the palatoplasty technique based on the patient’s characteristics, diagnostic workup results, knowledge, education, personal experience, and their previous positive and negative operative outcomes. As a result, a wide variety of surgical methods on the soft palate exist for the management of snoring and OSA. Palatal surgery for the management of snoring and OSA is a constantly evolving field. Sleep surgery is not new and is actually older than PAP therapy. However, every couple of years, a new approach or a modification is introduced. All of this increases the variability in sleep surgery practices within and between countries.

We established a panel of otolaryngology/head and neck experts in the surgical treatment of snoring and OSA to develop statements on decision-making regarding palatal surgery for treating snoring and OSA in adults. We initially aimed to reach a consensus on definitions and diagnoses regarding snoring and OSA [13]. In the second phase, the expert panel explored the agreement on definitions, patient criteria, and the diagnosis of snoring and OSA. We also assessed the level of agreement in decision-making and peri-operative considerations [14]. Finally, in this third phase, we had the goal of establishing the level of consensus among the experts regarding palatal surgery, its outcomes and follow-up, complications due to palatal surgery, and post-operative management after palatal surgery.

## 2. Materials and Methods

We established a panel consisting of eight European experts to develop consensus statements on diagnosing and treating snoring and obstructive sleep apnea (OSA). The foundational team of EO, ADV, and CV identified prominent otolaryngologists with an expertise in management of snoring and OSA, with a particular focus on palatoplasty. As a result, the expert panel consisting of 8 otolaryngologists was formed through invitation. The structured approach was outlined in our previous two manuscripts [13,14], and the modified Delphi method was utilized to explore the degree of consensus among these European experts [15,16,17,18]. These European experts practice in and represent 6 countries in Europe, including Poland, Italy, Belgium, Germany, Spain and the United Kingdom. The authors are all otolaryngologists who have been practicing in the field of sleep surgery for at least 2 decades.

Our previous work was focused on the fields of definitions and diagnosis [13] and decision-making in surgical management and peri-operative considerations [14]. This current work utilized the same methodology in relation to palatal surgery, outcomes and follow-up, complications, and post-operative management.

In summary, a thorough literature review was conducted by the first author (EO) to draft an initial list of statements. Statements were categorized into sections that later corresponded to the presented tables. Three authors (EO, ADV, CV) thoroughly reviewed, edited, and finalized the first set of statements, before they were distributed to all 8 panelists for their first round of responses. Each author was asked to mark that they either agreed or disagreed with each statement, to make comments if they so wished, and to suggest any revisions to the statement. As per the predefined methodology, the collected responses were collated on an Excel spreadsheet, and the rates of agreement and disagreement were calculated. The first author performed analysis of the responses and the feedback and modified the statements accordingly, marking the changes from the original. A file containing the revised set of statements with the summary statistics for each statement regarding the agreement rate was then tailored to each panelist to include each authors’ previous responses, the overall rates of agreement, and all deidentified comments for each statement, and requested each panelist to enter their new responses and any new comments or suggested edits, irrespective of what their previous response was. This process was performed over a total of 5 rounds. Following the established research protocol, for both the third and fourth iterations, a comprehensive process of reviewing panelists’ feedback and quantifying the degree of consensus was conducted. A renewed set of files were prepared and subsequently distributed to the panelists. For the culmination of this study, a definitive round of verification files were contrived by drawing attention to the responses that diverged from the majority.

For the current work, statements were categorized into sections related to palatal surgery, outcomes and follow-up, complications, and post-operative management. The initial set of statements was finalized and sent to the 8-member expert panel, which was established through invitation.

Panelists entered their decisions regarding their agreement or disagreement with each statement and provided comments or suggestions for modifications. After collecting all results and feedback, aggregated results with the counts and percentage of agreement or disagreement with each statement were calculated.

Aiming for a minimum of consensus rate of 75%, the second, third, and fourth rounds of circulation of the modified statements, calculation of results and comments, and new modifications were conducted. Finally, a verification file was sent to each panelist highlighting the statements representing the minority position among the eight panelists. After the verification round, the final set of statements was established.

The first author then drafted and circulated the manuscript among the panel members. Upon receiving edits and comments from each panelist, the final draft was circulated, and approval of all co-authors was obtained before submission.

## 3. Results

An initial set comprising 145 statements was disseminated to an expert panel to evaluate consensus regarding decisions regarding the surgical treatment of snoring and OSA. This panel, comprising eight members, evaluated each statement and marked that they were either in agreement or in disagreement with it.

In the primary evaluation phase, for the entire initial set of statements, the panelists registered their responses for an average of 72.8% of the disseminated statements. There was no response for 27.2% of the available statement options across all eight panelists.

A detailed examination of the entire initial set of statements revealed that 8.3% of the statements achieved unanimous consensus among all eight panelists. At this phase, 15.9% secured the consensus of seven panelists, 13.8% were supported by six panelists, 9% were agreed upon by five, and 11% were agreed upon four panelists. Furthermore, 9.7% and 10.3% of the statements found consensus among three and two panelists, respectively, while 15.9% received the endorsement of just one panelist and no statements faced unanimous rejection.

As described in our previous publication [13], the primary author analyzed the feedback, considering both the quantitative responses and the qualitative comments. Through this review, a refined set of statements was developed, which served as the second-round survey instrument. The revised document was organized to ensure clarity and precision as described in Part 1. Definitions and Diagnosis [13] and in Part 2. Decision-Making in Surgical Management and Peri-Operative Considerations [14]. Modified statements were marked and summary metrics were provided for each statement, detailing the number of panelists who had responded, as well as the count and percentage of those who agreed and disagreed with each statement.

The terminal set of statements focused on the themes of palatal surgery, outcomes and follow-up, complications, post-operative management related to snoring and Obstructive Sleep Apnea (OSA) comprised a total of 48 statements. An intricate examination of this set reveals that 27 of these statements were autonomous, whilst a subset of 21 statements stood as overarching themes, each further bifurcated into a total of 84 specific sub-statements. These sub-statements, delineated as “a”, “b”, “c”, and so forth, were intended to expound on the multifaceted aspects and intricacies of their respective parent statements. In summary, the final document on decision-making and peri-operative considerations included 111 autonomous or sub-statements. Starting with 145 statements and ending with 111 is a testament to how substantial the changes made in each round to the content and emphasis in the statements were. Due to the extensive revisions and consolidation of statements, it is not possible to calculate and present the correlation between the level of agreement between the rounds.

Within the segment delineated as palatal surgery for the treatment of snoring and OSA (Table 1), of the 34 statements, in the final round, a consensus was achieved among all eight panelists for 17 out of the 34 statements, equivalent to 50%. While panelists stated that local anesthesia could be utilized for certain palatoplasty techniques, they agreed that moderate to severe OSA required endotracheal general anesthesia. The goals of palate surgery were the advancement, expansion, and stiffening of tissues. All panelists agreed on the use of radiofrequency as the preferred ablation method. All panelists identified barbed stitch pharyngoplasty and expansion sphincter palatoplasty as both advancement and expansion methods. The panelists all agreed that DISE is essential in multi-level surgery.

**Figure 1:**
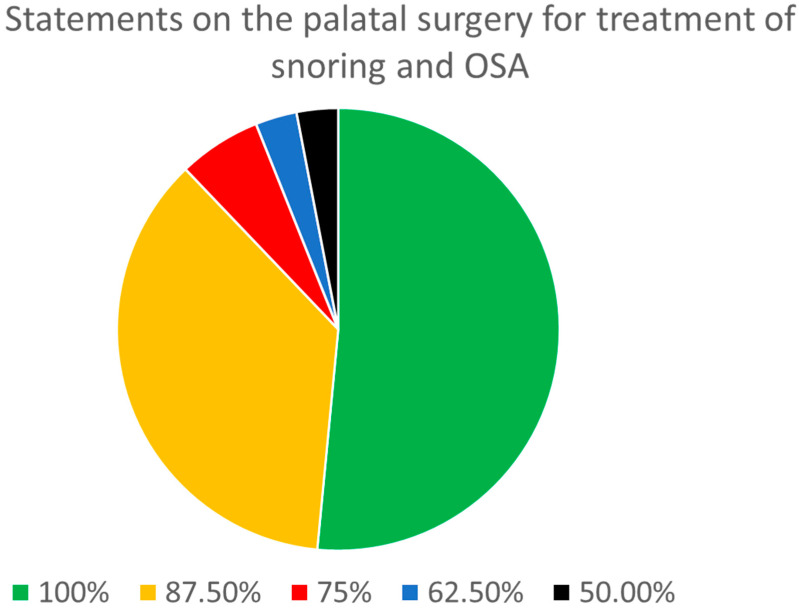
Distribution of the degree of consensus among panelists for the statements on palatal surgery for the treatment of snoring and sleep apnea.

Moreover, seven out of eight panelists achieved a consensus on 12 statements (35.3%) and six out of eight panelists agreed on 2 (5.9%) other statements out of the total of 34. Overall, of the 34 statements, 31 (91.2%) demonstrated a consensus among at least six (75%) of the panelists (Figure 1).

**Figure 2:**
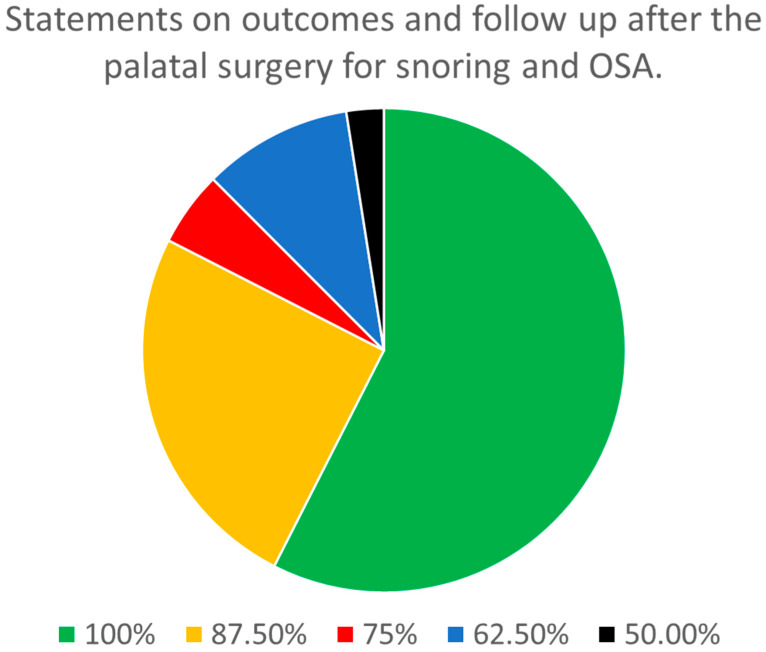
Distribution of the degree of consensus among panelists for the statements on outcomes and follow-up after palatal surgery for the treatment of snoring and sleep apnea.

Regarding the outcomes and follow-up after palatal surgery for the treatment of snoring and OSA (Table 2), there were a total of 43 statements. Of these, 12 statements were stand-alone, and 10 parent statements had 31 sub-statements. A consensus was reached among all eight panel members on 23 out of the 43 (53.3%) statements. The statements that achieved full consensus among the panelists included those related to the timing of follow-up, the use of symptom scores for outcomes, indications for repeating sleep studies after surgery and its timing, and definitions of failed surgery. Similarly, the need for a follow up for a minimum of 2 years, the need for a full re-evaluation in the case of failure, and the need for the consideration of different techniques or surgical sites were among the statements with full agreement. On 10 other statements (23.3%), there was agreement among seven of the panelists. On an additional two statements (4.7%), there was agreement among six of the panelists. Overall, of the 43 statements, 35 (81.4%) demonstrated a consensus among at least six (75%) of the panelists (Figure 2).

**Figure 3:**
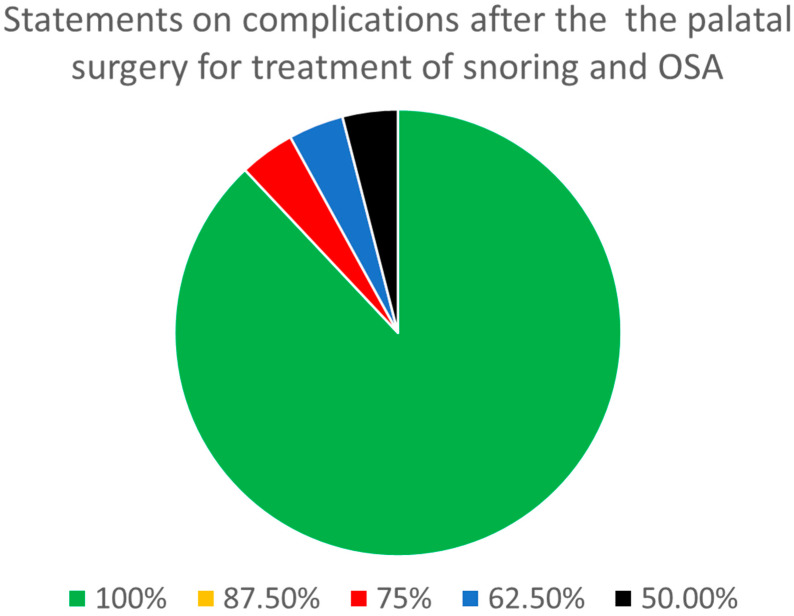
Distribution of the degree of consensus among panelists for the statements on complications after palatal surgery for the treatment of snoring and sleep apnea.

Regarding complications after palatal surgery for the treatment of snoring and OSA (Table 3), there were a total of 24 statements. Of these, two statements were stand-alone, and three parent statements had 22 sub-statements. A consensus was reached among all eight panel members on 22 out of the 24 (91.7%) statements. There was full agreement regarding all statements except for identifying difficulties during phonation among the perioperative complications and an intensive gag reflex among the early complications. There were no statements that showed agreement among seven of the panelists. On one additional statement (4.2%), there was agreement among six of the panelists. Overall, of the 24 statements, 23 (95.8%) demonstrated a consensus among at least six (75%) of the panelists (Figure 3).

**Figure 4:**
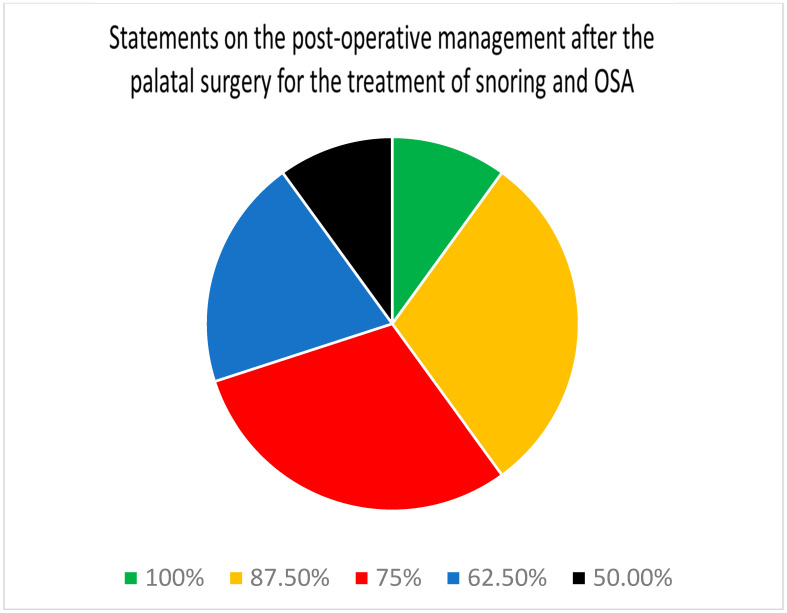
Distribution of the degree of consensus among panelists for the statements on post-operative management after palatal surgery for the treatment of snoring and sleep apnea.

Regarding post-operative management after palatal surgery for the treatment of snoring and OSA (Table 4), there were a total of 10 statements. Of these, one statement was stand-alone and two parent statements had nine sub-statements. Full consensus was reached among all eight panel members on only 1 out of the 10 (10%) statements related to pain management being essential for palatal surgery. On three other statements (30%), there was agreement among seven of the panelists. On an additional three statements (30%), there was agreement among six of the panelists. Overall, of the 10 statements, 7 (70%) demonstrated a consensus among at least six (75%) of the panelists (Figure 4).

## 4. Discussion

This paper reports on the efforts of a group of European experts in the field of sleep surgery to discuss statements on palatal surgery for the management of snoring and OSA. The goal was to assess and establish the level of consensus among the experts from various European countries with somewhat similar SDB prevalence, risk factors, education, socio-economic conditions, and availability of diagnostic tools and therapeutic options compared to the rest of the world. This effort is also expected to reflect differences in surgical concepts, preferences, and practices among sleep surgeons in European countries.

As discussed in our earlier work [1,2], the decision-making process in the surgical management of snoring and obstructive sleep apnea (OSA) has benefited significantly from methodological advancements such as the modified Delphi method. This method was employed to gather and synthesize the opinions of expert panel members, ensuring a robust consensus on the multifaceted aspects of OSA management.

The modified Delphi method ensured the anonymity of individual panelists, which was crucial to mitigating potential biases such as dominance or group conformity. This approach allowed for a more honest and uninfluenced exchange of ideas, fostering a diverse range of expert insights. Anonymity also facilitated the ease of modifications to proposed statements, enhancing the panelists’ ability to reach a higher degree of agreement without the pressure of conforming to dominant opinions.

The consensus derived from the modified Delphi method guides the development of clinical guidelines and best practices in surgical management. By incorporating a wide array of expert opinions, the resulting recommendations are more robust and reflective of the collective expertise within the field. This process also highlights the importance of considering individual patient factors, such as age, comorbidities, and response to previous treatments, in surgical decision-making.

Furthermore, the modified Delphi method is instrumental in identifying and prioritizing future research directions. By achieving consensus on areas of uncertainty and emerging challenges, this method helps to shape a focused and collaborative research agenda aimed at improving surgical outcomes for patients with OSA.

In the section related to palatal surgery for snoring and OSA, eight out of eight panelists reached a consensus on 17 out of the 34 statements. There was a consensus among at least six out of eight panelists regarding 31 statements (91.2%).

### 4.1. Palate as Target for Surgical Treatment of Snoring and OSA

All panelists agreed that palatoplasty is effective in treating snoring in most patients and in selected cases of OSA. Overall, 100% of panelists stated that palate surgery success depends on the patient and procedure selection. The goal of surgical treatment for snoring and/or OSA is to make changes to the anatomy and/or function that are thought to be responsible for snoring and/or OSA and, as a result, to improve these conditions. Before awareness and knowledge about OSA gradually improved in recent years, the focus was on snoring. The soft palate, particularly the uvula, has long been thought to be the source of vibration while breathing during sleep. Therefore, early surgical methods targeted reducing the volume of the tissues that vibrate. Later, these excess lax tissues in the soft palate were also targeted as the cause of upper airway obstruction [19,20].

From the surgical perspective, the soft palate, together with the tonsils and the uvula, became the primary point of attention due to the ability to examine its properties and its relative surgical accessibility. While tonsillectomy, once one of the most commonly performed surgeries, did improve snoring and OSA, it became clear later that there were other responsible tissues. Particularly for snoring, the main surgical targets were the uvula and the excess length of the soft palate. An awareness was cultivated that tonsillectomy alone does not often resolve OSA, mainly when the tonsils are not very large, especially in the adult population. Instead of performing tonsillectomy alone, surgical approaches were described that include the tonsils along with other concurrent manipulations of the soft palate tissues [21].

The initial surgery that was most commonly performed to treat snoring and OSA was uvulopalatopharyngoplasty (UPPP). The early outcomes of this procedure, particularly the limited success as well as the sequelae and subjective post-surgical complaints, resulted in the search for better surgical methods. Tang et al. reported, based on a review of 24 studies, that foreign body sensation occurred in 31.2% of patients, difficulty swallowing occurred in 17.7% of patients, dry pharynx occurred in 23.4% of patients, voice changes occurred in 9.5% of patients, and taste disturbances occurred in 8.2% of patients. The least common complication was velopharyngeal insufficiency (VPI), reported in 8.1% of cases one year after UPPP [22]. However, VPI was reported as the most common complication after this surgical procedure (28.5%) among patients that underwent UPPP with regard to their long-term evaluation of their quality of life [23]. Therefore, more effective, less invasive, and more functional palatal procedures were needed. With an better understanding of functional anatomy, various modifications of UPPP emerged, with variable satisfaction on behalf of the patients and surgeons. Later, a number of methods emerged, focusing on the ablation of specific tissue sites for the purpose of a reduction in volume. More recently, methods to stiffen the tissues, methods focusing on the re-alignment of tissues, and methods to change the muscle vectors have been introduced [24,25,26].

### 4.2. General vs. Local Anesthesia

A major factor affecting the selection of procedures on the soft palate is whether the procedure requires general anesthesia. In addition to the perceived risks of general anesthesia, as well as the allotted time and cost of the procedure, there have always been deterrent factors for both the patient and the surgeon. On the other hand, the compliance of patients with surgical manipulations and safety represented limited factors for procedures performed under local anesthesia. However, panelists displayed full agreement that certain palatoplasty procedures for snoring could be carried out under local anesthesia and 87.5% agreement regarding the use of local anesthesia procedures to treat mild OSA. There was complete consensus regarding the need for palate surgery under general anesthesia for moderate and severe OSA. These results are consistent with Friedman et al., who performed elevoplasties in an office setting and found it safe and effective for the treatment of snoring [27]. The study by Pang and Siow conducted a prospective, non-randomized trial on fifty-five patients with primary snoring and mild OSA who underwent bipolar radiofrequency palate reduction under local anesthesia. Overall, 77% percent of the patients reported a significant decrease in the mean snoring level, 83% reported an improvement in the Epworth Sleepiness Scale score, and 82% reported an improvement in their quality of life. The authors concluded that the performed procedures allowed them to achieve good results in snoring and mild sleep apnea [28]. O’Connor et al. performed a prospective study involving a combination of bipolar radiofrequency and injection snoreplasty under local anesthesia in 28 patients. This study showed that 360 days after the procedure, a reduction in snoring from 8.9 ± 0.7 to 4.1 ± 1.4 (*p* < 0.005) and in the AHI from 24.2 ± 7.1 to 12.8 ± 4.4 (*p* < 0.005) could be observed [29].

### 4.3. Role of DISE in Palatal Surgery

While DISE was not considered essential by all panelists prior to starting sleep surgery in all cases, the majority (87.5%) felt that it was essential before surgery on the palate. These results are consistent with those of Marzetti A et al. These authors performed DISE on 298 patients with primary snoring and OSA and stated that DISE is an important tool to assess the functional anatomy of the upper airways, the pattern of soft palate collapse and its severity, and epiglottic collapse and the nature of this collapse. DISE is helpful in making a personalized decision regarding the surgical technique [30]. De Vito A et al. also emphasized the necessity of obtaining a detailed description of the configurations of pharyngeal collapse and obstruction at all upper airway levels during DISE, which plays a crucial role in the decision-making for the treatment of OSA [31].

The selection of the surgical technique to be performed on the palate did not require DISE for 75% of the panelists. The panelists agreed that there was no need for a follow-up DISE in all patients. Elsobki et al. performed DISE only in the case of pharyngoplasty failure [32].

### 4.4. Goals of Palatal Surgery for Snoring and OSA

There was a consensus regarding the goals of palatal surgery for snoring and OSA, namely the advancement, expansion, and stiffening of the tissues. Apart from one panelist, all panelists agreed that the goal is not solely tissue ablation. This study demonstrates a high level of consensus on the indications for palatal surgery for OSA. This includes a consensus on the goals of palatal surgery, the preferred methods for each of the goals, and the personal preferences of the authors regarding each of the goals.

While most panelists (87.5%) defined modified UP3 and anterior pharyngoplasty as ablation methods, all agreed to recognize palatoplasty using radiofrequency as an ablation method, indicating that this is the preferred method. Bäck et al. published a systematic review of radiofrequency ablation of the soft palate to treat snoring to evaluate the effectiveness of and complications associated with this procedure. In that study, 30 articles out of the 159 identified met the inclusion criteria. Based on the results obtained from the included studies, the radiofrequency procedure applied to the soft palate did not cause severe adverse effects or postoperative pain. A decrease in snoring was observed in some of the studies [33]. The same first author conducted a randomized single-blinded placebo-controlled trial on 32 patients with mild OSA who received either a one-time palatoplasty using the radiofrequency technique or placebo under local anesthesia. The authors did not observe a significant difference between the two groups or a major decrease in OSA symptoms in the radiofrequency-treated group. Therefore, they do not recommend applying the radiofrequency procedure to the soft palate as a single-stage approach [34].

Barbed stitch pharyngoplasty (BRP) and expansion sphincter pharyngoplasty (ESP) were defined by all panelists as advancement methods. On the other hand, there was a lower level of agreement on defining other surgical methods as advancement procedures. Panelists disagreed on the preferred method of advancement palatoplasty.

All panelists agreed that ESP and BRP are expansion methods, and the majority (87.5%) agreed that lateral pharyngoplasty and pharyngoplasty with dorsal palatal flap expansion are also expansion methods. The level of agreement regarding the preferred method of expansion was 87.5% for ESP and 62.5% for BRP.

### 4.5. Single-Level vs. Multi-Level Surgery

All sleep surgery experts in the panel agreed that DISE is essential in performing multi-level surgery. The target sites for multi-level surgery were considered as the nose and base of tongue (87.5%). However, the nose alone (87.5%), the epiglottis alone (100%), and the base of the tongue alone (87.5%) were not considered common target sites for multi-level surgery. The panelists were all in agreement that in the presence of isolated obstruction and vibration, single-level palatoplasty was sufficient. Still, in the presence of multiple sites of obstruction and vibration, in addition to palatoplasty, other sites would need to be concurrently targeted.

### 4.6. Outcomes of Palatoplasty

Regarding outcomes and follow-up after palatal surgery for the treatment of snoring and OSA, eight out of eight panelists reached a consensus on 23 out of the 43 (53.5%) statements. There was a consensus among at least six out of eight panelists regarding 35 statements (81.4%).

There was 100% consensus among the expert panelists that palatoplasty is effective in treating snoring in most patients, but not in treating OSA in selected patients. Complete agreement was also observed on the fact that success could not be considered as certain and depended on the patient and the procedure selection. All panelists agreed that an early follow-up in 2–4 weeks was necessary to assess any complications.

All panelists also agreed on the need for a follow-up at least 2 months postoperatively to assess the outcomes. In studies conducted by Iannella et al. [35], Maniaci et al. [36], and Vicini et al. [37], the average postoperative follow-up was 6–6.8 months after barbed reposition pharyngoplasty was performed, while Tatla et al. used a follow-up of four months [38]. The latter conducted a prospective, non-randomized study on the effectiveness of radiofrequency palatoplasty in primary snorers. These authors evaluated different factors in different postoperative periods. For example, the median snoring score was evaluated and compared to the preparative baseline at 6 and 16 weeks following the initial treatment. The baseline mean Epworth Sleepiness Scale (ESS) score was compared to the ESS score obtained after 4 months. The effectiveness of radiofrequency-assisted palatoplasty performed under local anesthesia in the treatment of snoring was measured by Blumen et al. [39]. The authors evaluated the visual analog scale for snoring before and 2 months after the treatment.

Regarding outcomes and follow-up after palatal surgery for the treatment of snoring and OSA, eight out of eight panelists reached a consensus on 22 out of the 24 (91.7%) statements. There was a consensus among at least six out of eight panelists regarding 23 statements (95.8%).

All panelists agreed that following palatal surgery, site inflammation, edema of the palatal tissue, hematoma of the palatal tissue, and dysphagia may develop. The panelists also agreed that site inflammation is always a consequence of the procedure in the operated-upon areas. Iannella et al. observed swallowing problems for a few days in 11% of patients after palatal surgery, whereas 21% of patients were found to have dysphagia within the first 6 days postoperatively in the study of Montevecchi et al. [40,41].

Babademez et al. reported mild swallowing pain after barbed palatoplasty and expansion pharyngoplasty with anterior palatoplasty [42]. This pain resolved within a few days postoperatively. The outcomes of the study conducted by Iannella et al. indicated that a sore throat postoperatively was a common problem and was estimated to be severe in 36% of patients [40].

All panelists agreed with all early complications after palatal surgery besides an intensive gag reflex (62.5%). A dry pharynx was reported in 23.4% of patients in Tang’s et al. systematic review [22]. The most common sequela pointed out by the authors was related to foreign body sensation (31.2%). A sense of there being thick mucus in the throat can occur in up to 15% of patients who undergo barbed reposition pharyngoplasty.

The panelists reached 100% agreement that the following complications may occur or persist over 30 days postoperatively, i.e., a feeling of a foreign body while swallowing, speech disorders, increases in OSA severity, a sense of a foreign body in the throat, palatopharyngeal insufficiency, a sense of thick mucus in the throat, and nasopharyngeal stenosis. All of the above complications are widely described in the literature [23,24,25,37,43,44].

### 4.7. Post-Operative Management after Palatal Surgery

Regarding post-operative management after palatal surgery for the treatment of snoring and OSA, eight out of eight panelists reached a consensus on 1 out of the 10 (10%) statements. There was a consensus among at least six out of eight panelists regarding seven statements (70%).

Opioid analgesia in OSA patients has proven to be complex, as these patients are particularly prone to opioid-induced respiratory depression. Hence, it is essential to enhance postoperative monitoring to improve patient safety [45]. Waxman et al. supported the use of nonopioid analgesics and nonpharmacological approaches to pain management. These authors also proposed the use of preparatory local anesthetic infiltration and nonsteroidal anti-inflammatory drugs, and in cases where opioid medications are necessary, they recommended the use of tramadol or intranasal butorphanol [46].

## 5. Limitations

We are aware of the limitations of our study. First, eight European sleep surgeons were included in this study. A larger number of panelists would have been more representative. However, including non-sleep surgeons would lead to a lower level of consensus, and would not serve the purpose of providing a reference to other sleep surgeons. Second, the Delphi method requires multiple rounds, so the process was challenging and time-consuming. It also incurred the risk of including experts with similar points of view or who had conflicting interests, and did not represent every European country. However, there are a limited number of laryngologists whose primary practice is sleep surgery, so we believe that our panelists provide a well-balanced representation of the field of sleep surgery. Third, statements were changed based on the feedback obtained from each panelist, which led to a risk of disagreement with the revision from other panelists. However, among the 111 statements on palatal surgery for the treatment of snoring and OSA, outcomes and follow-up, complications, and the post-operative management of palatal surgery, full consensus was reached on 56.8% of them. Moreover, among all statements, consensus was achieved among at least six out of eight panelists on 86.5% of them. Therefore, we consider that we were able to reach a high level of agreement. A larger group of European panelists and modifications to the Delphi process may be considered in future projects.

## 6. Conclusions

Different palatal surgery techniques are used to treat snoring and OSA. The decision-making process for selecting a surgical method is complex and related to various factors on the part of the sleep surgeon and the country in which they practice. Differences among sleep physicians include their knowledge, education, and healthcare customs. Standards and practices are different between institutions, healthcare systems, and countries. Therefore, this consensus provides an overview of the work of European sleep surgeons to develop a set of statements on palatal surgery for the treatment of snoring and OSA, outcomes and follow-up, complications, and the post-operative management of palatal surgery. We believe that this will be helpful in everyday practice. It also indicates key areas for further studies in sleep surgery.

## Figures and Tables

**Table 1:** Statements on palatal surgery for the treatment of snoring and OSA. The percentage of consensus and number of panelists that agreed on statements are indicated with color codes.

Statements 100% (8) 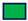 87.5% (7) 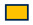 75% (6) 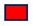 62.5% (5) 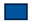 0% (4) 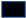	% Consensus	
1. Certain palatoplasty techniques for snoring can be performed under local anesthesia.	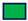	100
2. Certain palatoplasty techniques for mild OSA can be performed under local anesthesia.	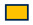	87.5
3. Palatoplasty for moderate or severe OSA should be performed under endotracheal general anesthesia.	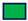	100
4. DISE is essential prior to starting of sleep surgery on the palate.	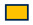	87.5
5. DISE is not essential prior to sleep surgery in all cases.		100
6. DISE is essential in deciding the type of surgery.	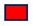	75
7. Follow-up DISE is not essential in all patients THAT UNDERGO palate surgery.	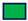	100
8. The goals of palate surgery ARE:		
a. NOT ablation of tissues	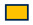	87.5
b. Advancement	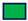	100
c. Expansion	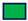	100
d. Stiffening	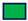	100
9. Ablation methods include:		
a. Modified uvulopalatopharyngoplasty (U3P)	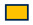	87.5
b. Radiofrequency ablation	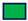	100
c. Anterior pharyngoplasty	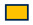	87.5
10. My preferred ablation method is radiofrequency ablation	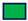	100
11. Advancement methods include:		
a. Australian modification of uvulopalatopharyngoplasty	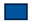	62.5
b. Barbed Stitch Resposition Pharyngoplasty (BRP)	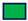	100
c. Lateral Pharyngoplasty (LP)	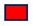	75
d. Transpalatal advancement	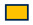	87.5
e. Espansion Sphincter Pharyngoplasty (ESP)	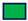	100
12. My preferred advancement method is Barbed Stitch Resposition Pharyngoplasty (BRP)	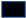	50
13. Expansion methods include:		
a. Espansion Sphincter Pharyngoplasty (ESP)	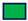	100
b. Barbed Stitch Resposition Pharyngoplasty (BRP)	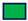	100
c. Lateral Pharyngoplasty (LP)	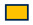	87.5
d. Pharyngoplasty with Dorsal Palatal Flap Expansion (PDPFEx)	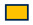	87.5
14. My preferred expansion method(s) is/are:		
a. Espansion Sphincter Pharyngoplasty (ESP)	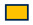	87.5
b. Barbed Stitch Resposition Pharyngoplasty (BRP)	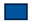	62.5
15. DISE is essential in multi-level surgery	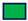	100
16. Besides palate, the most common sites of the upper airways for sleep surgery is:		
a. NOT nose ONLY	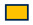	87.5
b. nose and base of tongue	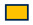	87.5
c. NOT epiglottis ONLY	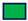	100
d. NOT base of tongue ONLY	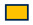	87.5
17. Single-level palatoplasty is sufficient if obstruction and vibration are related to one level of upper airways.	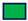	100
18. Multi-level sleep surgery in addition to palatoplasty is necessary if obstruction of and vibration are related to more than one level of upper airways.	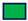	100

**Table 2:** Statements on outcomes and follow-up after palatal surgery for the treatment of snoring and OSA. The percentage of consensus and the number of panelists that agreed on statements are indicated with color codes.

Statements 100% (8) 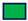 87.5% (7) 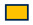 75% (6) 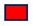 62.5% (5) 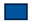 50% (4) 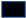	% Consensus	
1. Palatoplasty is effective for snoring in most patients.	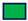	100
2. Palatoplasty is effective for OSA in SELECTED patients.	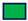	100
3. Palatoplasty success depend on patient and procedure selection.	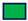	100
4. Palatoplasty success is not certain.	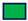	100
5. Post-operative follow-up for early complications and healing should be 2–4 weeks after the procedure.	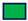	100
6. Post-operative follow-up for sleep surgery outcomes should be at least 2 months after the surgery.	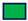	100
7. Patient-reported symptom scores are useful in assessing baseline snoring/OSA symptoms and treatment outcomes.	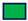	100
8. Patient reported symptom scores for assessing the surgical outcomes are:		
a. useful but not sufficient	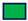	100
b. necessary	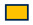	87.5
9. Patient reported quality of life questionnaires for assessment of surgical outcomes are		
a. useful	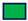	100
b. NECESSARY	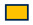	87.5
10. A repeat sleep study after a surgical procedure for OSA is essential	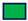	100
11 A repeat sleep study after a surgical procedure for OSA should be done if there is:		
a. moderate OSA	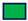	100
b. severe OSA, high BMI, presence of co-morbidities	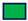	100
12. If needed, post-operative sleep study should be done:		
a. 3–5 months	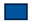	62.5
b. >6 months	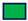	100
13. Definition of FAILED surgery is:		
a. no change and/or worsend OSA	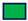	100
b. Less than 50% improvement in AHI	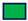	100
c. NOT CONSIDERED AS less than 50% improvement in AHI and AHI more than 20	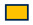	87.5
14. Definition of SUCCESSFUL surgery is:		
a. complete resolution of OSA	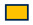	87.5
b. greater than 50% improvement in AHI	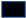	50
c. NOT “being able to use CPAP”	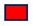	75
d. MORE than 50% improvement in AHI and AHI LESS than 20	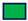	100
e. AHI LESS than 20	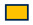	87.5
15. Even not considered failed palatal surgery, if there is still OSA:		
a. CPAP needs to be tried again, and wait for CPAP failure, prior to any consideration of surgical treatment	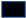	50
b. DISE needs to be repeated prior to any surgical treatment	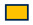	87.5
c. surgical treatment criteria and options MAY BE DIFFERENT FROM the initial surgery, THEY MAY BE MODIFIED based on the site and severity”	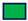	100
d. If the site of obstruction is still the palate, a repeat palatal surgery (with an alternative surgical method) MAY be performed	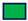	100
16. After failure of palatoplasty:		
a. CPAP needs to be tried again, and wait for CPAP failure, prior to any consideration of surgical treatment	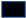	50
b. DISE needs to be repeated prior to any surgical treatment	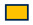	87.5
c. surgical treatment criteria and options MAY BE DIFFERENT FROM the initial surgery, THEY MAY BE MODIFIED based on the site and severity”	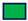	100
d. If the site of obstruction is still the palate, a repeat palatal surgery (with an alternative surgical method) will be performed	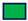	100
17. Patients with successful outcomes need continued follow- up for snoring and OSA.	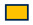	87.5
18. Patients with successful outcomes should be followed for minimum duration of:		
a. 1 year	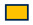	87.5
b. 2 years	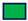	100
c. if patients/partners concern	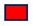	75
19. Patients with ongoing snoring / OSA after the surgery should undergo complete re-evaluation with the re-consideration of non-surgical treatment options	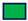	100
20. Repeat/alternative sleep surgery options should not be performed prior to 6 months after the first surgery:	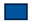	62.5
21. In case of just nasal surgery CPAP can be introduced 2–4 weeks after a nasal surgery	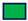	100
22. After the palate surgery for OSA, re-starting the use of CPAP:		
a. SHOULD NOT START immediately IF/WHEN MOS drops below 90%”	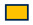	87.5
b. MAY BE STARTED after 2 months postoperatively IF/when no satisfying success related to snoring and sleep parameters	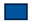	62.5
c. SHOULD NOT START immediately, the same day	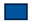	62.5
d. MAY BE STARTED after 2 weeks	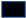	50

**Table 3:** Statements on complications after palatal surgery for the treatment of snoring and OSA. The percent of consensus and the number of panelists that agreed on statements are indicated with color codes.

Statements 100% (8) 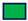 87.5% (7) 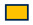 75% (6) 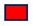 62.5% (5) 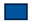 50% (4) 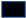	% Consensus	
1. Perioperative complications include:		
a. site inflammation	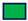	100
b. oedema of palatal tissue	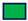	100
c. hematoma of the palate	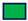	100
d. difficulties while swallowing	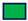	100
e. difficulties during phonation	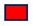	75
2. Early (up to 30 days postoperatively) complications include:		
a. site inflammation	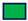	100
b. post-tonsillectomy hemorrhage (2–5% of patients).	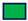	100
c. throat dryness	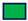	100
d. palatal hematoma	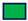	100
e. impaired taste	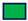	100
f. difficulties during fonation	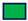	100
g. intensive gag reflex	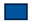	62.5
h. a sense of a foreign body in the throat	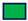	100
i. a sense of thick mucous in the throat.	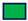	100
3. Late (after 30 days postoperatively) complications include:		
a. feeling of the foreign body while swallowing	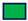	100
b. swallowing disorders	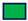	100
c. speech disorders	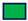	100
d. increase in OSAS severity	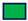	100
e. sense of a foreign body in the throat.	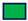	100
f. palatopharyngeal insufficiency	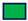	100
g. sense of thick mucous in the throat.	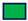	100
h. MAY INCLUDE nasoplaryngeal stenosis	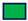	100
4. Postoperative pain after palatoplasty that require pain management may last for 2 weeks	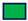	100
5. The most severe postoperative pain is reported by patients on the 5–7th day	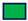	100

**Table 4:** Statements on the post-operative management after palatal surgery for the treatment of snoring and OSA. The percentage if consensus and the number of panelists that agreed on statements are indicated with color codes.

Statements 100% (8) 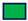 87.5% (7) 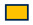 75% (6) 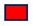 62.5% (5) 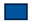 50% (4) 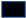	% Consensus	
1. Pain management is essential for patients undergoing palatal surgery.	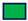	100
2. The most frequently used pain medication options are:		
a. Paracetamol (up to 4.0 g/day)	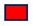	75
b. Paracetamol (up to 4.0 g/day) with Metamizolum (up to 3.0 g/day)	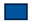	62.5
c. If the above pain medication is not sufficient, Tramadol (up to 400 mg/day).	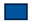	62.5
d. Steroids orally Dexamethasone 2 mg for 5 days	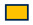	87.5
e. Mouthwash: Benadryl 1 teaspoon, Magnesium hydroxide 1 table spoon, 4% lidocaine 1 table spoon-gargle 3 times per day	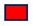	75
f. 24–48 h i.v. perfusion of morphine followed by paracetamol	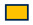	87.5
g. throat spray with lidocaine over the counter	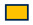	87.5
3. Prophylactic antibiotics for patients undergoing palatal surgery		
a. is necessary during the surgery	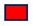	75
b. is necessary post-operatively	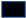	50

## Data Availability

Data are contained within the article.
